# Enablers and Barriers of Zinc Fortification; Experience from 10 Low- and Middle-Income Countries with Mandatory Large-Scale Food Fortification

**DOI:** 10.3390/nu13062051

**Published:** 2021-06-15

**Authors:** Ann Tarini, Mari S. Manger, Kenneth H. Brown, Mduduzi N. N. Mbuya, Laura A. Rowe, Frederick Grant, Robert E. Black, Christine M. McDonald

**Affiliations:** 1 Independent Consultant, Laval, QC H7G 3Z5, Canada; 2 International Zinc Nutrition Consultative Group, University of California, San Francisco, CA 94143, USA; mari.manger@izincg.org (M.S.M.); Christine.mcdonald@ucsf.edu (C.M.M.); 3 IZiNCG Fortification Task Force, San Francisco, CA 94143, USA; khbrown@ucdavis.edu (K.H.B.); mmbuya@gainhealth.org (M.N.N.M.); laura.rowe@ffinetwork.org (L.A.R.); frederick.grant@gatesfoundation.org (F.G.); rblack1@jhu.edu (R.E.B.); 4 Department of Nutrition, Institute for Global Nutrition, University of California, Davis, CA 95616, USA; 5 Global Alliance for Improved Nutrition, Washington, DC 20036, USA; 6 Food Fortification Initiative, Atlanta, GA 30322, USA; 7 Helen Keller International, New York, NY 10017, USA; 8 Institute for International Programs, Johns Hopkins Bloomberg School of Public Health, Baltimore, MD 21205, USA; 9 Department of Pediatrics, San Francisco School of Medicine, University of California, San Francisco, CA 94143, USA

**Keywords:** large-scale food fortification, zinc, qualitative study, enablers, barriers, LMIC, micronutrients, nutrition policy, undernutrition

## Abstract

Adequate zinc nutrition is important for child growth, neurodevelopment, immune function, and normal pregnancy outcomes. Seventeen percent of the global population is estimated to be at risk for inadequate zinc intake. However, zinc is not included in the fortification standards of several low- and middle-income countries with mandatory fortification programs, despite data suggesting a zinc deficiency public health problem. To guide policy decisions, we investigated the factors enabling and impeding the inclusion of zinc as a fortificant by conducting in-depth interviews with 17 key informants from 10 countries. Findings revealed the decision to include zinc was influenced by guidance from international development partners and enabled by the assessment of zinc deficiency, mandatory regional food fortification standards which included zinc, the World Health Organization (WHO) guidelines for zinc fortification, and the low cost of zinc compound commonly used. Barriers included the absence of zinc from regional fortification standards, limited available data on the efficacy and effectiveness of zinc fortification, and the absence of national objectives related to the prevention of zinc deficiency. To promote zinc fortification there is a need to put the prevention of zinc deficiency higher on the international nutrition agenda and to promote large-scale food fortification as a key deficiency mitigation strategy.

## 1. Introduction

Adequate zinc nutrition is important for ensuring the growth and neurodevelopment of children, healthy immune function, and normal pregnancy outcomes [1]. By analyzing national data on the prevalence of child stunting and the availability of zinc in the food supply, Wessells and Brown have estimated that 17.3% of the world’s population is at risk of inadequate zinc intake [2]. While therapeutic zinc supplementation is recommended for the treatment of childhood diarrhea [3], less attention has been directed to interventions to prevent and/or mitigate zinc deficiency as part of micronutrient deficiency control policies and programs in low- and middle-income countries (LMICs).

Large-scale food fortification (LSFF; henceforth referred to as fortification) is the mandatory or voluntary addition of essential micronutrients to widely consumed staple foods and condiments during production [4]. Fortification is one of the main strategies recommended for enhancing dietary zinc intake among nutritionally vulnerable populations alongside bio-fortification, dietary diversification, and supplementation. It is considered one of the most cost-effective strategies to improve micronutrient intake with the advantage of delivering nutrients to large segments of the population without requiring changes in food consumption patterns [4,5]. LSFF with iodine, vitamin A, iron, and folic acid leads to improvements in the micronutrient and health status of women and children in LMICs [6]. Moreover, a recent systematic review found that zinc fortification reduces the prevalence of zinc deficiency and may reduce the incidence of diarrhea [7].

Many food vehicles have been mandated for fortification in LMICs following a process of first identifying gaps in the dietary intake of micronutrients and then identifying fortifiable staple foods and/or condiments frequently consumed in sufficient quantities by the target population that have proven to be adequate carriers of the missing nutrients. The national fortification mandates include the specific amounts and types of the missing nutrient to be added to the food during food processing based on the unique nutritional needs of the country. As of April 2021, 114 LMICs have mandatory fortification of at least one staple food (i.e., wheat flour, maize flour, edible oil, rice, salt) in place, of which 72 have mandatory fortification programs for at least one kind of cereal grain (wheat flour, maize flour, or rice). Yet, out of these 72 countries, only 29 are known to have a standard that includes zinc as a mandatory fortificant [8,9] even though more of these countries could benefit from zinc fortification.

The aim of this study was to understand factors that enabled or hindered the inclusion of zinc in national fortification programs and to assess other contextual factors related to fortification programming in a country that may affect the inclusion of zinc. The specific objectives were to identify: (1) key reasons and data behind the decision to consider including zinc as a fortificant in LMICs where zinc deficiency was identified as a public health problem; (2) the main barriers and/or challenges preventing countries from including zinc as a fortificant; (3) how zinc fortification is impacted by national micronutrient policies and strategies; (4) how zinc is or could be considered in activities assessing compliance, coverage, and impact of fortification; and (5) how the cost of zinc fortification may have impacted the decision to include zinc.

## 2. Materials and Methods

### 2.1. Country Selection

**Figure 1:**
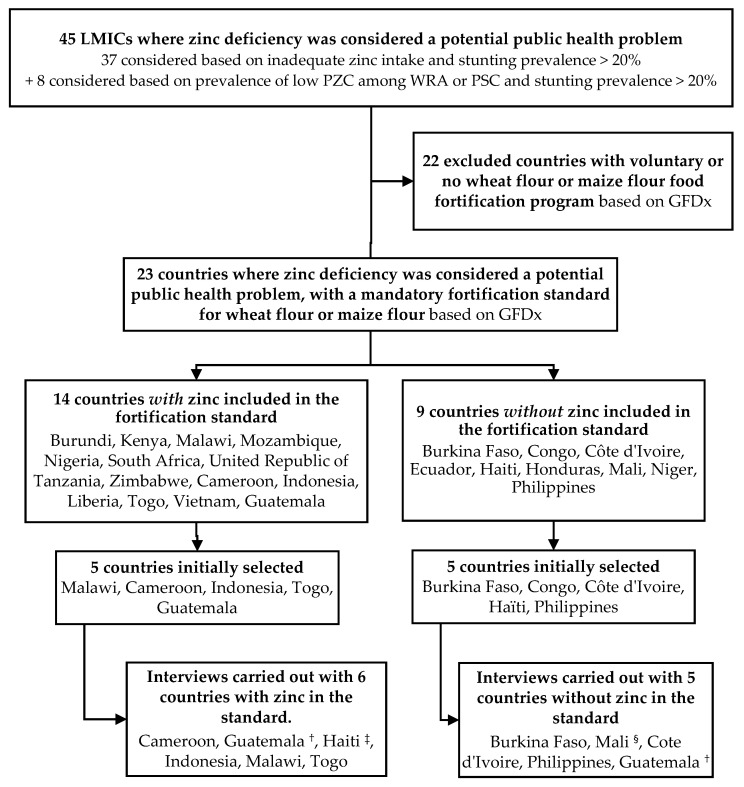
Country selection process. ^†^ Guatemala fortifies maize flour, but not wheat flour, with zinc; ^‡^ Haiti was initially selected as a country not fortifying with zinc but turned out to indeed be fortifying with zinc; ^§^ Mali replaced the Congo due to difficulties identifying a key informant in Congo. LMIC: low- and middle-income countries; PZC: plasma zinc concentration; PSC: preschool children; WRA: women of reproductive age.

To guide the country selection process for this study, a matrix was developed using the supplemental table in Wessells and Brown [2] that lists all LMICs with an elevated risk of inadequate zinc intake based on food balance sheet data. This country database was then updated with (1) the most recent estimates of the prevalence of stunting from the WHO/United Nations Children’s Fund (UNICEF)/World Bank database [10] and (2) the prevalence of low plasma zinc concentration (PZC) in preschool children (PSC) and/or women of reproductive age (WRA) as reported in the WHO Micronutrient Database [11] and/or the 2017 publication by Hess [12]. Zinc deficiency was considered a potential public health problem if the country had a high risk of inadequate zinc intake and a prevalence of stunting ≥20%; or a high prevalence of low PZC in PSC or WRA and a prevalence of stunting ≥20%. Based on this methodology, zinc deficiency was identified as a potential public health problem in 23 countries where mandatory wheat or maize flour fortification was in place according to the Global Fortification Data Exchange database (GFDx) online tool [8]. Of these, 14 countries included zinc as a fortificant and 9 did not. From that list, we selected 5 countries where zinc was included as a mandatory fortificant and 5 countries where zinc was not included as a mandatory fortificant to gain greater insight into national decisions to include or exclude zinc. The total number of countries identified for further exploration was limited to 10 to allow for in-depth interviews with key informants and because this number was considered sufficient to allow for representation from different geographical regions with different official languages and a variety of food fortification development partners. Based on information available in the GFDx, Malawi, Cameroon, Indonesia, Togo, and Guatemala were selected as zinc fortifying countries, meaning that they had at least one mandatory flour fortification program with zinc in the standard. Burkina Faso, the Republic of the Congo, Côte d’Ivoire, Haïti, and the Philippines were selected as non-zinc fortifying countries because zinc was not on the list of fortificants included in the standard. However, following the start of the interview process, the Congo was replaced by Mali due to difficulties in identifying a key informant, and Haiti was found to have been initially misclassified because details of the micronutrients in the standard were not yet published and thus not registered in the GFDx. During the interview, Haiti was determined to be zinc fortifying and moved to that group; Guatemala was considered to be in the zinc fortifying category because it was fortifying maize flour with zinc (but not wheat flour). However, the interviews revealed rich information about the difference between the processes for wheat and maize fortification programs, and, therefore, Guatemala was classified in both categories. The country selection process is illustrated in Figure 1.

### 2.2. Development of the Interview Guide

In parallel to the country selection process, two interview guides were developed for zinc fortifying and non-zinc fortifying countries. The interview guides both included a list of questions about decisions made at key stages of the LSFF program cycle (i.e., problem description/definition; policy formulation; program design; and program implementation, monitoring, and evaluation). Some questions covered the food fortification program in general, to identify factors that influenced the decision about micronutrients to include in the standard and allow for possible unforeseen enablers or challenges to emerge from the discussions. Specific questions related to the decision to include or exclude zinc as a fortificant were formulated according to the zinc fortification situation in the country. Some questions about the implementation of zinc fortification (e.g., about the cost of the zinc compound or the implication of zinc fortification for the monitoring system) were asked only to the zinc fortifying countries.

### 2.3. Key Informant Selection and Planning of the Interviews

Food fortification partners in each country were identified to solicit their support in identifying and contacting appropriate in-country informants. We looked for individuals involved in the design and/or implementation of the program and representation from various sectors (i.e., government, supporting partners, consultants, and research institutions). The interviewer was either introduced to the potential respondent by an intermediary partner or contacted the informant directly. Once contact was established, the interviewee was provided with the interview guide in advance to assist in preparation for the interview and to ensure a more complete understanding of the structure and content of the interview and informed consent procedure. Country informants who could provide complementary information were solicited for additional responses. To mitigate the risk of losing the spontaneity of responses, the shared guide was a summary version and during the introduction to the interview, it was reiterated that the exercise was to learn about the experience from each country.

### 2.4. Data Collection: Constraints, Difficulties, and Adjustments Made to the Methodology

This study used an iterative research process, whereby ongoing data analysis informed minor adjustments to the methodology in terms of the selection of countries (Section 2.1) and informants, and the content of the interview guide. The 10 core key informant interviews were conducted between February and June 2020 by the first author. The process of identifying the appropriate interviewees was complicated by the COVID-19 pandemic, which made it difficult to interact with stakeholders given work disruptions, competing priorities, and the difficulties inherent in conducting virtual interviews. Group interviews and/or interviews with multiple individuals within a country were attempted but proved impractical given scheduling challenges. Consequently, one informant was interviewed per country, except in three countries (Philippines, Mali, and Togo). Five informants representing the Philippines contributed by email. Two informants from a development partner organization in Mali consulted with each other and contributed responses. These informants also had previous experience with Mali’s food fortification program as in-country staff. Two informants representing Togo collectively shared a policy and historical point of view combined with a past and current program implementation perspective. All individual informants contributed information on all stages of the program cycle, but with different levels of depth according to experience.

Semi-structured interviews were conducted using the GoToMeeting platform (LogMeIn, Boston, MA, USA) and took approximately one hour to complete. For two countries (Mali and Indonesia), interviews were conducted via email due to problematic internet connections. For the Philippines, information was collected by email from a group of stakeholders.

Interviews/email exchanges were conducted in French or English, depending on the informant’s preferred language. Detailed notes were taken by the interviewer during live interviews and email responses were consolidated into one document per country.

### 2.5. Review of Additional Resources

Details about national zinc fortification levels and other fortified vehicles were extracted from the (GFDx) in preparation for the interviews. Several additional documents identified by key informants were reviewed following the interviews, including national food fortification alliance terms of references, regulatory documents, survey reports, meeting reports, and other project documents. These documents complemented the information received during interviews and facilitated further analysis and comparison across countries.

### 2.6. Data Synthesis and Analysis

Data were analyzed using a thematic analysis approach. An initial list of themes was generated from topics in the interview guides based on the stages of the policy/program cycle. This allowed for the identification of enablers and barriers at specified stages in the cycle. Some themes that emerged during the interviews were further pursued in subsequent interviews and emails for greater clarification (e.g., the influence of regional standards and zinc compound used if not specified in the GFDx). Notes from interviews were summarized and classified in an Excel spreadsheet by country and theme. Responses under each theme were then compared among countries including or not including zinc to identify differences. Common issues affecting national fortification programs in general were also captured since these challenges could influence the potential impact of current or future zinc fortification programs. A select number of quotes were extracted from interview notes and from emails to illustrate the ideas expressed in the analysis. Prominence was given to views expressed by national/governmental stakeholders.

## 3. Results

A total of 16 informants from the 10 countries participated in the interviews and email exchanges. Interviewees represented a variety of backgrounds: eight were from supporting development partner organizations, three from research institutions, three from ministries of health, and three were independent consultants with expertise in food fortification. Most stakeholders had occupied a variety of positions in different organizations over the course of the fortification program cycle, supporting the program in different ways over time. For example, three development organization informants and one consultant had formerly worked for the ministry of health during the early years of the program. One informant had previously represented the public health national laboratory within the national food fortification alliance. Eight informants continued to have an active role in the food fortification program at the time of the interview: two within the ministry of health, two as consultants, and four within development organizations. Informants responded to questions regarding the various roles they played at different stages of the program. Most informants were involved at the onset of the program while two became involved a few years after program initiation. The depth of information provided was dependent on the interviewee’s knowledge of the program and the length of time and period he/she was associated with the program. All participants were based in the country for which they were interviewed. We were not able to reach representatives from national standards bureaus or from compliance control institutions. However, information collected from interviewees and documents covered all stages of the program cycle.

Because the influence of the regional standard was mentioned by all the informants from West African countries, the regional fortification advisor was contacted to learn more about the process for establishing the regional standard, including the decision to make zinc an optional micronutrient in the West African Economic and Monetary Union (UEMOA) standards. Additionally, two country informants played a role in and provided information on the creation of regional standards: one for the East African Community (EAC) and the other one for five countries in Central America.

### 3.1. Fortification Program Characteristics of Participating Countries

Fortification program characteristics for the ten countries are described in Table 1. The date of enactment of mandatory fortification varied from 2000 to 2017. Wheat flour was the dominant vehicle with only two countries adding zinc to maize flour (Malawi and Guatemala). Malawi mandated the fortification of both wheat and maize flour while Guatemala mandated the fortification of maize flour only. In countries where the type of zinc fortificant was specified in the national standard, zinc oxide was listed in all countries but Guatemala, which requires zinc bisglycinate. National country standards for zinc ranged from 32% to 159% of the WHO recommended zinc level based on extraction, fortificant compound, and estimated per capita flour availability [13].

### 3.2. Considerations Behind the Decision to Include (or Not Include) Zinc as a Fortificant

#### 3.2.1. Guidance from Experts and Development Partners

In all studied countries, food fortification discussions were initiated by a development partner, including non-governmental organizations, United Nations agencies, research institutes, and bilateral agencies projects, either nationally as part of a food fortification project or as part of a regional initiative. Development partners usually provided technical and financial support for advocacy and start-up activities such as surveys, capacity assessments, capacity building, and standards development. They often engaged consultants to collect information required for project design. Thus, in many countries, the decisions about the micronutrients to include in the fortification program were very much influenced by external partners and their expert consultants’ guidance.

“It is (the partner) who told us to include zinc, and based on information provided, we decided it was good to make it mandatory”.

(Informant from Togo)

In zinc-fortifying countries, partners (and hired expert consultants) shared and disseminated zinc fortification information from the WHO guidelines, supported assessments of national risk of zinc deficiency, and/or supported baseline micronutrient surveys, thus identifying and raising the issue of zinc deficiency as a public health problem.

#### 3.2.2. Assessment of Zinc Deficiency

In most zinc-fortifying countries, partners supported country assessments of the risk of zinc deficiency based on proxy indicators such as empirical knowledge of dietary patterns, documented iron deficiency, and/or the prevalence of child stunting. Some informants cited the parallels with iron deficiency, for example, diets that included small amounts of animal-source foods and that relied primarily on grain and legume staples high in phytates.

Three countries with mandatory zinc fortification (Guatemala, Malawi, and Cameroon) conducted national micronutrient surveys that assessed dietary zinc intake and PZC around the time of the start of the program. Guatemala waited for nationally representative survey data before including zinc in its maize flour fortification standard. However, in Malawi and Cameroon, the decision to include zinc as a fortificant was taken before the survey results were available and were based solely on empirical knowledge of dietary patterns. With that said, from the informants’ perspective, the availability of detailed dietary data and documented zinc deficiency based on biomarker data from the surveys did help guide program design and subsequent impact assessment. For example, in Cameroon, a 2009 national micronutrient survey [14] assessed dietary intake and PZC for women and young children. The dietary intake data helped confirm the choice of wheat flour as a food vehicle and the fortification level for multiple micronutrients including zinc. Dietary data were later used to model the possible impact of wheat flour fortification on zinc intake, including the reduction in the proportion of various target populations with insufficient zinc intake. Nationally, the prevalence of zinc deficiency based on low PZC was 76.9% in women and 69.1% in children, which confirmed suspected deficiency and served as a baseline for comparison with future survey results.

The availability of nationally representative data on zinc status did not always translate into zinc being included in the fortification standard. In the Philippines, two national nutrition surveys that measured PZC in the population found a prevalence of zinc deficiency of 30% in 2008 and 26% in 2013, indicative of a public health problem [15]. However, this information did not trigger changes to the fortification standard, with one informant stating that additional studies justifying zinc fortification were deemed necessary.

“Adding zinc as a fortificant is possible because the law allows the (public nutrition body) to add nutrients to be covered by mandatory food fortification. But (…) the reviewers of a policy recommendation would want to see a range of studies done—technology (how to fortify in the chosen food vehicle), acceptability, stability, and efficacy ...”.

(Informant from the Philippines)

#### 3.2.3. Regional Fortification Standards

Regional fortification standards guided several countries in Africa and the Americas to include or not include zinc in their fortification standards. In the East, Central and Southern Africa (ECSA) Health Community, which includes 14 countries, and in the East African Community (EAC), which includes six countries, zinc was included in respective regional guidelines for wheat flour fortification resulting in most member countries adopting zinc fortification in their national standards. When zinc was an optional component in the West African (UEMOA) regional wheat flour fortification standard covering eight countries, only one country included it. In Central American, the harmonized regional fortification standard for wheat flour adopted in 2002, which covers five countries, did not include zinc fortification, neither as mandatory nor voluntary. Even after survey findings showed that zinc deficiency constituted a public health problem in 2010, Guatemala did not fortify wheat flour with zinc since the inclusion of zinc would have required a modification to the regional fortification standard which would have required initiating a lengthy process among all five counties in the region.

#### 3.2.4. The Lack of Evidence of the Efficacy and Effectiveness of Zinc Fortification

The lack of evidence around the efficacy of zinc fortification in reducing the prevalence of zinc deficiency was mentioned by some countries as a barrier to zinc fortification. During the elaboration of the West Africa regional wheat flour standard, zinc was considered but made optional after a study showed no effect of a zinc-fortified complimentary food on plasma zinc concentration of young children in Senegal [16]. Subsequently, only one UEMOA country made zinc fortification mandatory.

Another informant from Guatemala reported that flour producers were requesting “proof” of the impact of the fortification program on the nutritional status of the population, as well as information on the bioavailability of the zinc compound.

### 3.3. Considerations for Sustainable Implementation of a Food Fortification Program with or without Zinc

#### 3.3.1. Available Guidelines and Tools for Food Fortification

The WHO/FAO Guidelines on Food Fortification with Micronutrients [4] informed the design of many fortification programs and were used by development partners when advising on the inclusion of and appropriate levels for zinc fortification. Five countries mentioned using the guidelines at the onset of the program, although one eventually decided not to mandate zinc fortification because zinc was not a mandatory fortificant in the regional standard. While the WHO guidelines help facilitate the inclusion of zinc in some countries, this was not universal. Indeed, the guidelines had been published when fortification legislation was passed in the four West African countries, three of which did not include zinc in their fortification standard. In Indonesia, fortification with zinc was initiated before the publication of the WHO/FAO Guidelines. Because of concerns about possible adverse impact on iron bioavailability, the initial intended level of zinc fortification was revised downward. The adopted amount corresponds to 32% of the WHO-recommended level assuming that zinc oxide is used by all producers.

One informant reported the importance of guidance from the international scientific community, and the lack of specific guidance for zinc, at the time when the fortification program was put in place in his country (in the late 1990s, early 2000s).

“The nutrients of interest were vitamin A and iron (iodine too, of course, but salt iodization was done earlier) (…) because of the high prevalence of vitamin A deficiency and anemia as indicated in the (National Nutrition Survey) and the possible consequences documented in the International Vitamin A Consultative Group (IVACG) and International Nutritional Anemia Consultative Group (INACG) publications, etc. The International Zinc Nutrition Consultative Group (IZiNCG) came much later”.

(Informant from the Philippines)

#### 3.3.2. Industry Trials to Assess Changes in Organoleptic Properties or Shelf-Life

Three informants in countries fortifying with zinc reported conducting shelf life and acceptability trials with the food industry. Findings from these trials indicated no significant differences when compared to non-fortified flour, which enabled acceptance of zinc fortification. However, in Guatemala, after an initial trial showed no effect of the premix (including zinc) on maize flour properties, producers reported that the original premix formulation adversely affected the color of the product made with maize flour over time. Even though zinc was not specifically identified as the component affecting the color, both the iron and zinc levels in the premix were revised downward, followed by regulatory changes.

#### 3.3.3. Cost of Zinc Fortification

According to informants, the cost specifically pertaining to the addition of zinc as a fortificant was perceived as negligible by food producers and in-country premix producers. The only exception was in Guatemala, where the cost of zinc bisglycinate was an issue raised by the maize flour and premix producers, partly due to the small quantity that was purchased on an annual basis. However, producers continued to fortify maize flour with this compound. Zinc fortification was not specifically impacting the cost of monitoring since only iron was used as a proxy for all micronutrients included in the premix.

#### 3.3.4. Zinc—the Lost New Kid on the Block

Recognition of zinc deficiency as a public health problem by food fortification stakeholders and inclusion of zinc in fortification programs did not always translate into broader zinc deficiency control policy objectives or zinc being discussed by the nutrition community outside food fortification platforms. Zinc deficiency appeared to “get lost”, either entirely or under the umbrella of “prevention of micronutrient deficiencies” in national nutrition plans and therefore was not on the agenda of meetings coordinating implementation or monitoring progress of those plans. Zinc was, however, recognized for the treatment of diarrhea in all 10 countries and this aspect was integrated into country plans.

Discussions about micronutrient deficiency prevention in many countries continued to revolve around iron, vitamin A, and iodine, and to a lesser extent, folate. These better-known deficiencies were already a concern when flour fortification started in all 10 countries, and these micronutrients were naturally considered as possible fortificants. Wheat/maize flour fortification primarily interested ministries of health for its potential to contribute to the reduction in iron deficiency and anemia in a cost-effective way and was viewed as complementary to existing interventions such as iron-folic acid supplementation of pregnant women.

“The main consideration was on contributing iron to people’s diet as iron deficiency is a national nutrition problem in the country. The other micronutrients (zinc, vitamin B1, vitamin B2, and folic acid) were basically “opportunistic” when they were included in the fortificant premix with the following considerations: (i) they are very cheap; (ii) they are deficient in people’s diet.”

(Informant from Indonesia)

#### 3.3.5. Overall Food Fortification Program Performance

Once the decision was made to include zinc as a fortificant and the program was launched, the impact of zinc fortification was very much dependent on the performance of the fortification program overall. The phasing out of partner financial and technical support coupled with insufficient national ownership of the fortification program was often cited as a reason for the lack of sufficient resources for coordination and compliance monitoring activities by the government in countries fortifying or not fortifying with zinc. Many informants believed that recurring costs for monitoring activities could be included in the national budget if there was greater awareness of the advantages of food fortification among high-level authorities. Enablers and barriers to mandatory zinc food fortification are summarized in Table 2.

### 3.4. Findings Related to Program Monitoring and Evaluation

#### 3.4.1. Regulatory Monitoring/Surveillance

Regulatory monitoring refers to activities conducted by an authorized government department(s) to monitor domestic or imported food adherence to a national standard. None of the countries fortifying with zinc mentioned quantitative testing of the level of zinc fortification as part of their routine regulatory monitoring of fortified flour. Instead, iron was quantitively tested when samples were collected, and the level of the other nutrients was inferred based on the composition of the premix used by industries per global guidance. The presence of the fortification logo on the packaging was also mentioned as a means of monitoring if products were fortified on the market.

#### 3.4.2. Coverage Assessment of Fortified Foods

Among countries fortifying with zinc, only the Cameroon informant mentioned conducting coverage surveys post fortification. In Cameroon, wheat flour samples were collected from households and markets in the two major cities (Yaoundé and Douala) one year after wheat flour fortification began to assess the coverage of fortified food. Data showed that 76% of collected wheat flour samples were considered fortified (≥13 mg Zn/kg) and the mean zinc content of fortified samples (94 mg total Zn/kg) was close to the mandated national standard (95 mg added Zn/kg) while the mean zinc level of pooled samples from households and markets was 78% of the zinc national standard [17]. However, a second monitoring activity carried out 5 years after program initiation in the 10 regions of Cameroon and each of the two major cities showed a lower mean zinc content of pooled market and household samples representing 45% of the zinc standard. Survey results were used to advocate for reinforcement of the regulatory monitoring system [18].

#### 3.4.3. Micronutrient Biomarker Surveys Pre- and Post-Zinc Fortification

Of the six countries fortifying with zinc, only Cameroon had pre- and post-zinc fortification data on zinc status; plasma zinc and other biomarkers of micronutrient status were measured in Douala and Yaoundé one year after initiation of fortification [17]. The survey was limited to those cities due to resource limitations, and the likely availability of fortified products and the relatively high consumption of wheat flour observed there during the baseline survey. Compared to baseline, results showed that the prevalence of low PZC concentrations dropped from 46.8% to 28.4% among preschool children, and from 39.4% to 21.6% among women of reproductive age in the two cities. These differences persisted after adjusting for age, breastfeeding status (children), pregnancy (women), malaria, inflammation, household characteristics, and variables related to the timing of blood sampling.

Across all countries, micronutrient biomarker surveys were perceived as costly and difficult to obtain funding for, even when nutrition stakeholders uniformly expressed the need for the data.

## 4. Discussion

LSFF is increasingly recognized as a cost-effective approach to improve population micronutrient status and related health outcomes [6,7,19]. This study identified factors that inhibited countries with evidence of an elevated potential risk of zinc deficiency from introducing zinc as a fortificant, as well as factors that facilitated countries to include zinc in their fortification program. The identification of these factors will be useful to support the adoption or reinforcement of zinc fortification in countries where populations could benefit the most from this intervention. The main factors that enabled the inclusion of zinc in national food fortification programs were: the advice from development partners supporting the program, direct or indirect assessment of the national prevalence of zinc deficiency, mandatory regional food fortification standards which included zinc, the WHO guidelines for zinc fortification and, the low cost of zinc oxide. The main factors that impeded the inclusion of zinc in the national food fortification program were: the absence of advice regarding zinc from international development partner supporting the program, the absence of zinc in regional food fortification standards, lack of evidence of efficacy and effectiveness of zinc fortification, the lack of awareness of the national prevalence of zinc deficiency, and the lack of zinc deficiency prevention objectives in national and international policies and dialogues. In some countries with zinc fortification, the absence of international guidelines on zinc fortification at the time of the launch of the program, trials showing adverse effects of fortification on organoleptic properties of the final product without identifying which compound affected the properties, or concerns of adverse effects on iron bioavailability, led to the revision of the zinc fortification level downward.

The strong influence of development partners on the decision to include zinc in food fortification programs was a consistent finding throughout the interviews. Food fortification partners and experts more often advised countries to consider zinc as a fortificant in new programs in recent years compared with countries where discussions to design a fortification program were initiated 10 or more years ago. This is indicative of a growing awareness of zinc deficiency as a public health problem [12,20] and the growing availability of guidance and tools to support countries with zinc fortification and standards development [4]. With more evidence on the impact of zinc fortification on health outcomes, the existence of relevant resources and tools on zinc fortification, and an increasing number of country experiences to learn from, there is an opportunity to support countries where zinc deficiency is likely to be a public health problem to update their food fortification standards to include zinc.

The documentation of zinc deficiency as a likely public health problem was an important step in the inclusion of zinc in national fortification standards. However, only one of the six interviewed countries having zinc as a fortificant based their decision to include zinc on actual zinc status assessments. All other five countries accepted proxy indicators such as dietary intake patterns. Most of the countries where zinc was not included as a fortificant lacked data to estimate the potential risk of deficiency (proxy or PZC) or were not aware of the existence of such data. This implies that when assessing the need for zinc fortification in countries, proxy indicators may be acceptable when biomarker data is not available. However, LMICs should aim to assess the burden of zinc deficiency using PZC prevalence and/or inadequate zinc intake with a nutritional and/or health survey to establish a baseline [9] that could be used for assessing the impact after the launch of zinc fortification to track progress and ensure ongoing ownership and acceptance of the program by the public and private sector.

Regional fortification standards for trade zones also guided national fortification programs in some countries, including the decision to include zinc and the optimal levels of fortification. However, when regional standards excluded zinc, it became even more challenging for countries to add it later. Additionally, regional standards often provide a minimum level to facilitate effective trade in a region; these regional standards are not adapted to each country’s consumption patterns or zinc intake levels. Therefore, regional standards should allow flexibility for countries to modify their fortification standards to go beyond the minimum and/or add additional micronutrients per the needs of each country.

In many countries, fortification programs suffered from a decline in performance after the initial support from development partners ceased. Coverage and quality gaps in LMIC were also reported in recent publications [21,22]. In some cases, this could be due in part to development partner support which was focusing primarily on the development of legislation and capacity building of industry to fortify and of control bodies to monitor, with less attention paid to building long-term governance capacity and ensuring sustainable financing. The latter aspects should be addressed when discussing zinc fortification to ensure sustainable impact.

For countries where zinc deficiency is likely to be a public health problem, zinc did not appear prominently on the nutrition agenda when compared with iron, vitamin A and iodine. Even when zinc was included in fortification programs, its effect on dietary zinc intake or biochemical status was not specifically monitored by the program nor in broader nutrition coordination mechanisms. This may be due to the absence of specific zinc deficiency reduction objectives in national nutrition strategies and plans, which are often aligned with international objectives (e.g., Sustainable Development Goals and World Health Assembly nutrition objectives) in addition to poor visibility of the LSFF agenda as part of the broader multisectoral nutrition agenda [22]. Absence of zinc in the United Nations micronutrient priorities, despite its deficiency being recognized as a major risk factor in the global burden of disease, has previously been identified as an issue hindering efforts towards international health objectives [23].

Our findings indicate that sustainable zinc fortification in LMICs requires more effective anchoring in national nutrition plans and the development of comprehensive zinc deficiency reduction strategies including LSFF. Considering the influence of development partners on national nutrition and of international health objectives and priorities, a call to action should be made to the international nutrition community to prioritize the prevention of zinc deficiency and to reinforce the importance of zinc in national fortification programs as an effective strategy for the improvement of zinc status in high burden countries [7,24]. A specific communications strategy is needed to build awareness, mobilize resources, and strengthen the capacity of development partners and national nutrition stakeholders including relevant government agencies and national fortification coordination bodies. Practical zinc-specific guidance, advocacy materials, and tools using up-to-date data should be made available to international and national nutrition stakeholders to support this purpose. Countries where zinc deficiency is considered a potential public health problem and where mandatory fortification is in place but zinc is not included in the standard should be prioritized for further support to consider the inclusion of zinc. Countries in the same category with non-existent or voluntary fortification programs should be supported to determine whether they could potentially benefit from a mandatory zinc fortification program assuming that they have appropriate food vehicles to carry the fortificant to the segments of the population at greatest risk of zinc deficiency.

To promote zinc fortification, there is a need to put zinc deficiency prevention higher on international and national nutrition agendas and to promote zinc fortification as part of an effective and sustainable component of a deficiency mitigation strategy.

## Figures and Tables

**Table 1:** Characteristics of national mandatory wheat and/or maize flour fortification programs in 10 selected countries where zinc deficiency is considered a potential public health problem.

Country Category	Country	Year of Mandatory Legislation	Vehicle	Compound	Zinc Fortification Level	% of Recommended Fortification Level [13]
**Countries with Mandatory Food Fortification Program Including Zinc**	Malawi	2015 2015	Wheat flour Maize flour	zinc oxide zinc oxide	80 mg/Kg 40 mg/Kg	159% ^†^
	Cameroon	2011	Wheat flour	zinc oxide	95 mg/Kg	100%
	Haiti	2017	Wheat flour	Unspecified in current legislation.	60 mg/Kg	63% ^‡^
	Indonesia	2001 (updated in 2009, 2018)	Wheat flour	Unspecified ^§^	30 mg/Kg	32% ^‡^
	Togo	2012	Wheat flour	zinc oxide	55 mg/Kg	58%
	Guatemala	2016	Maize flour	zinc bisglycinate	15 mg/Kg	N/A ^¶^
**Countries with Mandatory Food Fortification Program not Including Zinc**	Guatemala	2002	Wheat flour	N/A	N/A	N/A
	Burkina Faso	2012	Wheat flour	N/A	N/A	N/A
	Côte d’Ivoire	2007 (updated in 2018)	Wheat flour	N/A	N/A	N/A
	Mali	2012	Wheat flour	N/A	N/A	N/A
	Philippines	2000	Wheat flour	N/A	N/A	N/A

^†^ calculated for the two vehicles in combination using WHO recommendations for zinc addition levels. Food and Agriculture Organization (FAO) estimated per capita flour availability for 2013 was used for the calculation. ^‡^ Assuming that zinc oxide is used by all producers. ^§^ The zinc compound is unspecified in the 2009 mandatory standard in force but the 2018 standard, not yet gazetted, specifies zinc oxide as the compound. ^¶^ There is no WHO recommendation for this zinc compound. N/A: Non-applicable.

**Table 2:** Summary of enablers and barriers to mandatory zinc food fortification.

Enablers	Barriers
Advice from supporting development partners and experts to consider zinc as a potential fortificant. Assessment of the national zinc deficiency burden. Regional food fortification standards that include zinc as a mandatory fortificant. WHO guidelines for zinc fortification. Industry trials showing no adverse effects on fortified product properties. Low cost of zinc oxide. No additional zinc-specific cost to the monitoring system.	Absence of guidance from supporting development partners for zinc fortification. Regional food fortification standards not having zinc as a mandatory fortificant. Lack of international guidelines for zinc fortification at the time of the launch of the program. Lack of evidence on the efficacy and effectiveness of zinc fortification. The absence of zinc deficiency prevention in national and international nutrition agendas. Poor national commitment and/or capacity to maintain the food fortification program.

## Data Availability

Not applicable.

## References

[1] Brown K., Rivera J., Bhutta Z., Gibson R., King J., Lonnerdal B., Ruel M., Sandtröm B., Wasantwisut E., Hotz C. (2004). International Zinc Nutrition Consultative Group (IZiNCG) Technical Document #1. Assessment of the Risk of Zinc Deficiency in Populations and Options for Its Control. Food Nutr. Bull..

[2] Wessells K., Brown K. (2012). Estimating the Global Prevalence of Zinc Deficiency: Results Based on Zinc Availability in National Food Supplies and the Prevalence of Stunting. PLoS ONE.

[3] World Health Organization (2004). Clinical Management of Acute Diarrhoea: WHO/UNICEF Joint Statement.

[4] Allen L.H., De Benoist B., Dary O., Hurrell R. (2006). World Health Organization Guidelines on Food Fortification with Micronutrients/Edited by Lindsay Allen.

[5] Horton S., Alderman H., Rivera J.A. The Challenge of Hunger and Malnutrition; Copenhagen Consensus: 2008. https://www.copenhagenconsensus.com/sites/default/files/cp_hungerandmalnutritioncc08vol2.pdf.

[6] Keats E.C., Neufeld L.M., Garrett G.S., Mbuya M.N.N., Bhutta Z.A. (2019). Improved Micronutrient Status and Health Outcomes in Low- and Middle-Income Countries Following Large-Scale Fortification: Evidence from a Systematic Review and Meta-Analysis. Am. J. Clin. Nutr..

[7] Tsang B.L., Holsted E., McDonald C.M., Brown K.H., Black R., Mbuya M.N.N., Grant F., Rowe L.A., Mager M.S. (2021). Effects of Foods Fortified with Zinc, Alone or Co-Fortified with Multiple Micronutrients, on Health and Functional Outcomes: A Systematic Review and Meta-Analysis. Adv. Nutr..

[8] Global Fortification Data Exchange (GFDx). https://fortificationdata.org/list-of-countries-for-the-food-fortification-dashboard/.

[9] Gupta S., Brazier A., Lowe N. (2020). Zinc Deficiency in Low- and Middle-Income Countries: Prevalence and Approaches for Mitigation: Zinc Deficiency in Low- and Middle-Income Countries. J. Hum. Nutr. Diet..

[10] UNICEF, WHO (2019). The World Bank Joint Child Malnutrition Estimates—Levels and Trends.

[11] WHO Micronutrient Database. https://www.who.int/vmnis/database/en/.

[12] Hess S.Y. (2017). National Risk of Zinc Deficiency as Estimated by National Surveys. Food Nutr. Bull..

[13] World Health Organization (2009). Recommendations on Wheat and Maize Flour Fortification. Meeting Report: Interim Consensus Statement.

[14] Helen Keller International Cameroun, Ministère de la Sante Publique du Cameroun (2011). National Survey of Micronutrient Status and Consumption of Fortifiable Foods.

[15] Food and Nutrition Research Institute-Department of Science and Technology (FNRI-DOST) (2015). Philippine Nutrition Facts and Figures 2013: 8th National Nutrition Survey/Biochemical Survey.

[16] Lo N.B., Aaron G., Hess S., Idohou-Dossou N., Guiro A., Wade S., Brown K. (2011). Plasma Zinc Concentration Responds to Short-Term Zinc Supplementation, but Not Zinc Fortification, in Young Children in Senegal. Am. J. Clin. Nutr..

[17] Engle-Stone R., Nankap M., Ndjebayi A.O., Allen L.H., Shahab-Ferdows S., Hampel D., Killilea D.W., Gimou M.-M., Houghton L.A., Friedman A. (2017). Iron, Zinc, Folate, and Vitamin B-12 Status Increased among Women and Children in Yaoundé and Douala, Cameroon, 1 Year after Introducing Fortified Wheat Flour. J. Nutr..

[18] Mark H.E., Assiene J.G., Luo H., Nankap M., Ndjebayi A., Ngnie-Teta I., Tarini A., Pattar A., Killilea D.W., Brown K.H. (2019). Monitoring of the National Oil and Wheat Flour Fortification Program in Cameroon Using a Program Impact Pathway Approach. Curr. Dev. Nutr..

[19] Olson R., Gavin-Smith B., Ferraboschi C., Kraemer K. (2021). Food Fortification: The Advantages, Disadvantages and Lessons from Sight and Life Programs. Nutrients.

[20] Black R.E., Victora C.G., Walker S.P., Bhutta Z.A., Christian P., de Onis M., Ezzati M., Grantham-McGregor S., Katz J., Martorell R. (2013). Maternal and Child Undernutrition and Overweight in Low-Income and Middle-Income Countries. Lancet.

[21] Osendarp S.J.M., Martinez H., Garrett G.S., Neufeld L.M., De-Regil L.M., Vossenaar M., Darnton-Hill I. (2018). Large-Scale Food Fortification and Biofortification in Low- and Middle-Income Countries: A Review of Programs, Trends, Challenges, and Evidence Gaps. Food Nutr. Bull..

[22] Mkambula P., Mbuya M.N.N., Rowe L.A., Sablah M., Friesen V.M., Chadha M., Osei A.K., Ringholz C., Vasta F.C., Gorstein J. (2020). The Unfinished Agenda for Food Fortification in Low- and Middle-Income Countries: Quantifying Progress, Gaps and Potential Opportunities. Nutrients.

[23] Gibson R. (2006). Zinc: The Missing Link in Combating Micronutrient Malnutrition in Developing Countries. Proc. Nutr. Soc..

[24] Shah D., Sachdev H., Gera T., de Regil L., Pena-Rosas J. (2016). Fortification of staple foods with zinc for improving zinc status and other health outcomes in the general population. Cochrane Database Syst. Rev..

